# Disentangling the links between gastric emptying and binge eating *v*. purging in eating disorders using a case-control design

**DOI:** 10.1017/S0033291721003640

**Published:** 2023-04

**Authors:** Pamela K. Keel, Lisa A. Eckel, Britny A. Hildebrandt, Alissa A. Haedt-Matt, Daryl J. Murry, Jonathan Appelbaum, David C. Jimerson

**Affiliations:** 1 Department of Psychology, Florida State University, Tallahassee, FL, USA; 2 Department of Psychology and Neuroscience Program, Florida State University, Tallahassee, FL, USA; 3 Department of Psychiatry, University of Pittsburgh School of Medicine, Pittsburgh, PA, USA; 4 Department of Psychology, Illinois Institute of Technology, Chicago, IL, USA; 5 College of Pharmacy, University of Nebraska Medical Center, Omaha, NE, USA; 6 College of Medicine, Florida State University, Tallahassee, FL, USA; 7 Department of Psychiatry, Beth Israel Deaconess Medical Center, Boston, MA, USA

**Keywords:** Purging disorder, Bulimia Nervosa, binge eating, gastric emptying, medication

## Abstract

**Background:**

Prior work supports delayed gastric emptying in anorexia nervosa and bulimia nervosa (BN) but not binge-eating disorder, suggesting that neither low body weight nor binge eating fully accounts for slowed gastric motility. Specifying a link between delayed gastric emptying and self-induced vomiting could offer new insights into the pathophysiology of purging disorder (PD).

**Methods:**

Women (*N* = 95) recruited from the community meeting criteria for DSM-5 BN who purged (*n* = 26), BN with nonpurging compensatory behaviors (*n* = 18), PD (*n* = 25), or healthy control women (*n* = 26) completed assessments of gastric emptying, gut peptides, and subjective responses over the course of a standardized test meal under two conditions administered in a double-blind, crossover sequence: placebo and 10 mg of metoclopramide.

**Results:**

Delayed gastric emptying was associated with purging with no main or moderating effects of binge eating in the placebo condition. Medication eliminated group differences in gastric emptying but did not alter group differences in reported gastrointestinal distress. Exploratory analyses revealed that medication caused increased postprandial PYY release, which predicted elevated gastrointestinal distress.

**Conclusions:**

Delayed gastric emptying demonstrates a specific association with purging behaviors. However, correcting disruptions in gastric emptying may exacerbate disruptions in gut peptide responses specifically linked to the presence of purging after normal amounts of food.

Delayed gastric emptying has been observed in numerous gastrointestinal diseases marked by increased postprandial fullness, nausea, and stomach discomfort (Hajishafiee, Bitarafan, & Feinle-Bisset, 2019; McClain, Humphries, Hill, & Nickl, 1993) and was originally investigated and supported as an explanation of these symptoms in anorexia nervosa (AN) (Hadley & Walsh, 2003; Robinson & McHugh, 1995). Like AN, purging disorder (PD) is characterized by increased postprandial gastrointestinal distress (Keel, Wolfe, Liddle, DeYoung, & Jimerson, 2007; Keel et al., 2018a), and this has been posited as contributing to its core symptom of purging in the absence of binge eating (Keel & Striegel-Moore, 2009). Unlike AN, PD occurs in individuals of minimally healthy weight [i.e. body mass index (BMI) ⩾18.5 kg/m^2^], leaving unclear whether findings in AN would extend to those with PD. Supporting that findings may extend to PD, several independent studies found delayed gastric emptying in bulimia nervosa (BN) (Devlin et al., 1997; Geliebter et al., 1992; Inui et al., 1995; Kamal et al., 1991; Kiss et al., 1990), which occurs in individuals of minimally healthy weight who binge and use inappropriate compensatory behaviors. Further, Walsh, Zimmerli, Devlin, Guss, and Kissileff (2003) noted an association between slower gastric emptying and more frequent vomiting in BN. Other studies have reported no differences between BN and control participants (Hutson & Wald, 1990; Koch, Bingaman, Tan, & Stern, 1998; Robinson, Clarke, & Barrett, 1988), particularly when BN participants were not required to use self-induced vomiting as a compensatory behavior. In addition, delayed gastric emptying has not been found in patients with binge-eating disorder (BED) (Geliebter, Yahav, Gluck, & Hashim, 2004). Similar to patients with BN, those with BED have large out of control binge-eating episodes, but they do not use inappropriate compensatory behaviors. Therefore, none use self-induced vomiting. This pattern of results suggests that vomiting, rather than low weight or binge eating, may be directly related to delays in gastric emptying in eating disorders. Specifying a link between slowed gastric motility and self-induced vomiting could provide novel insight into biological contributions to PD – an Other Specified Feeding or Eating Disorder for which more information is needed to understand and treat the presence of purging in the absence of binge eating.

The current study examined gastric emptying in women with PD and BN who used vomiting as their primary purging method, women with nonpurging BN, and non-eating disorder controls to determine whether (i) gastric emptying is linked to binge eating, purging, or both, (ii) gastric emptying is associated with postprandial gastrointestinal distress, and (iii) a medication that increases gastric motility impacts differences in gastric emptying and postprandial gastrointestinal distress. Data from participants in the current study were included in prior analyses to examine whether postprandial peptide YY (PYY) and ghrelin responses contributed to differences in subjective responses to a fixed test meal (Keel et al., 2018a) and whether a behavioral measure of satiation using an *ad lib* meal confirmed self-reported responses to a fixed meal (Keel et al., 2018b). Data from these participants were also included in secondary analyses unrelated to the aims of the parent project (Davis, Smith, & Keel, 2020; Forney, Crosby, Brown, Klein, & Keel, 2021; Keel, Bodell, Haedt-Matt, Williams, & Appelbaum, 2017; Maske, Williams, & Keel, 2020). This is the first report of gastric emptying and of responses to the fixed meal in the medicated condition from this sample.

## Methods

### Participants

Women (*N* = 95), 18–45 years old, were recruited from the community in Iowa City, IA from 2008 to 2009 and Tallahassee, FL from 2009 to 2013 to participate in a four-visit study. The current paper focuses on women who completed all four visits, representing 80% of the 119 eligible women who initiated the study. Demographic and clinical variables, including diagnostic group, did not differ significantly by recruitment site or study completion, with one exception; those recruited from Florida endorsed greater impairment related to their eating disorder (*p* = 0.049). All participants had a BMI between 18.5 and 26.5 kg/m^2^, were free of current mood or substance use disorders based on diagnostic interviews, not pregnant or nursing within the past 6 months, medication free, and free from medical conditions that could influence weight or appetite based on a medical screen. Hormonal contraceptives for the purpose of contraception were permitted. To dissociate the effects of binge eating *v.* purging, women were recruited if they met inclusion criteria for one of four groups: (1) DSM-5 BN with purging (BNp); (2) DSM-5 BN using only nonpurging compensatory behaviors (BNnp); (3) research criteria for PD (Keel & Striegel-Moore, 2009); or (4) healthy controls with no history of eating disorders and no current weight loss behaviors. All individuals in the BNp and PD groups used self-induced vomiting as their primary purging behavior. Groups did not differ significantly on age [mean (s.d.) = 20.8 (2.0) years; *F*_(3, 91)_ = 0.11, *p* = 0.96], BMI [mean (s.d.) = 22.7 (1.9) kg/m^2^; *F*_(3, 91)_ = 0.12, *p* = 0.95], or ethnic/racial background [χ^2^ (9) = 5.04, *p* = 0.83] or percent White, non-Hispanic [χ^2^ (3) = 1.09, *p* = 0.79]. Racial/ethnic composition was 76% white, non-Hispanic, 12% African American, 5% Hispanic, and 7% Asian.

The authors assert that all procedures contributing to this work comply with the ethical standards of the relevant national and institutional committees on human experimentation and with the Helsinki Declaration of 1975, as revised in 2008. This study was approved by the Human Subjects Committee of the IRB at both institutions where data were collected, and participants provided written informed consent prior to study participation. Participants were paid $75, $50, $100, and $100 across the four visits.

### Procedure

During their first visit, participants completed a medical exam, including pregnancy tests and tests of liver function, the Structured Clinical Interview for DSM-IV Axis I Disorders (SCID) (First, Spitzer, Gibbon, & Williams, 1995), the Eating Disorders Examination (EDE) (Fairburn & Cooper, 1993), and self-report questionnaires (see Table 1) to establish eligibility and characterize clinical features across groups. Proposed DSM-5 criteria were available at the time of study initiation and applied. Interrater reliability was high in the current study (*κ* = 0.90 for EDE eating disorder diagnosis, *r* > 0.97 across EDE subscales, and *κ* > 0.90 for current and lifetime SCID diagnoses). During their second visit, participants completed an *ad lib* test meal as a behavioral assay of satiation, and data from this visit are reported elsewhere (Keel et al., 2018b).

**Table 1.** Descriptive statistics for clinical measures and gastric emptying by group

	Control (*n* = 26)	PD (*n* = 25)	BNnp (*n* = 18)	BNp (*n* = 26)
Measures (*α*)	Mean (s.d.)	Mean (s.d.)	Mean (s.d.)	Mean (s.d.)
EDE total (0.96)	0.07 (0.08)	3.27 (0.85)	3.41 (0.94)	4.06 (0.91)
Restraint (0.88)	0.05 (0.17)	3.79 (1.34)	3.43 (1.34)	4.13 (1.33)
Eating concern (0.77)	0.00 (0.00)	1.37 (1.13)	1.94 (1.14)	2.73 (1.27)
Weight concern (0.89)	0.11 (0.15)	3.62 (1.19)	3.93 (1.08)	4.62 (0.87)
Shape concern (0.92)	0.10 (0.11)	3.62 (0.96)	3.75 (1.00)	4.35 (0.99)
TFEQ				
Restraint (0.94)	3.43 (2.21)	16.42 (4.69)	15.28 (5.73)	16.62 (3.70)
Disinhibition (0.90)	2.12 (1.37)	7.59 (3.88)	11.61 (2.48)	12.27 (2.32)
Hunger (0.89)	2.62 (1.70)	5.73 (3.93)	9.27 (3.72)	9.14 (3.71)
BSQ (0.99)	39.85 (5.83)	132.88 (27.21)	129.06 (31.65)	155.80 (24.82)
BDI (0.91)	0.88 (1.56)	10.08 (6.14)	11.28 (6.51)	16.81 (9.62)
STAI				
State (0.96)	24.77 (4.76)	40.48 (13.37)	40.67 (10.15)	45.48 (14.80)
Trait (0.95)	27.19 (5.98)	42.85 (10.78)	46.15 (7.98)	51.30 (12.64)
Impairment				
CIA (0.96)	0.15 (0.61)	15.93 (8.54)	21.94 (8.94)	28.04 (9.58)
GAF	90.77 (4.32)	61.84 (6.55)	63.50 (6.06)	61.64 (5.73)
Gastric emptying AUC (μg/ml × min)	313.64 (163.09)	253.25 (114.05)	266.94 (116.56)	209.35 (106.10)

BDI, Beck Depression Inventory (Beck, Ward, Mendelson, Mock, & Erbaugh, 1961); BSQ, Body Shape Questionnaire (Cooper, Taylor, Cooper, & Fairburn, 1987), CIA, Clinical Impairment Inventory (Bohn et al., 2008); EDE, Eating Disorder Examination (Fairburn & Cooper, 1993); GAF, Global Assessment of Function (First et al., 1995); STAI, State-Trait Inventory (Spielberger, Gorsuch, Lushene, Vagg, & Jacobs, 1983); TFEQ, Three Factor Eating Questionnaire (Stunkard & Messick, 1985); AUC, Area Under the Curve.

During the third and fourth visits, participants came to the lab between 7:30–8:00 h after an overnight fast to complete the standardized meal assessment used in our prior study (Keel et al., 2007). An intravenous catheter was placed in their arm, they rested for 5 min and then were given a pill to consume with a small amount of water by a registered nurse. The pill was either a single 10 mg dose of metoclopramide or a placebo. Prior work supported the safe use of a 10 mg oral dose of metoclopramide in conjunction with 1.5 g of acetaminophen to assess gastric emptying (Boivin, Carey, & Levy, 2003; van Wyk, Sommers, Meyer, & Moncrieff, 1990). All participants completed the test meal assessment under both conditions, with condition order determined by block randomization conducted by the first author (PKK). Neither participants nor the research staff running visits knew whether the pill contained metoclopramide or placebo. One hour after taking the pill, two fasting blood samples (5 ml) were drawn 10 min apart and averaged to measure preprandial gut-peptide concentrations. Participants consumed 900 kCal of Ensure Plus^®^, with 1.5 g of acetaminophen dissolved in the liquid meal, immediately after the second fasting blood sample. Acetaminophen is absorbed into the blood stream after it enters the intestinal track, permitting valid and reliable indirect assessment of gastric emptying of liquid meals through repeated measures of postprandial plasma concentrations of acetaminophen (Sanaka, Kuyama, & Yamanaka, 1998). This method has been used safely in numerous studies (Willems, Quartero, & Numans, 2001), including studies of eating disorders (Geliebter et al., 2004), and offers the advantage of not exposing participants to radioactive materials used in scintigraphy (Hens et al., 2017). Participants were instructed to not take any medications, including over-the-counter medications within 72 h of their third and fourth visit and informed of the acetaminophen dose in each meal and the importance of not exceeding the daily recommended limit for this medication. Postprandial blood draws occurred 10, 20, 30, 50, 90, and 120 min to measure acetaminophen concentrations and gut peptide responses. Gastric emptying rate was assessed by changes in plasma acetaminophen concentrations from 10 to 50 min postprandially, corresponding to peak associations between plasma concentrations of the acetaminophen tracer and direct assessment of gastric emptying of a liquid meal (Koizumi, Kawamura, Ishimori, Ebina, & Satoh, 1988; Sanaka et al., 1998). After each sample, participants completed Visual Analog Scales (VAS) to capture momentary changes in subjective experiences. Momentary ratings of ‘nausea’ and ‘stomach ache’ scores were averaged to create a composite gastrointestinal distress score (*α* = 0.85) (Keel et al., 2018a, 2018b). Visits were separated by ⩾48 h to prevent carry-over effects.

### Gastric emptying assessment

Plasma acetaminophen concentrations were measured using a validated Liquid Chromatography-Mass Spectrometry (LCMS) method (Baliga & Kallury, 2008). Briefly, plasma (200 μl) was spiked with internal standard (phenacetin 10 μg/ml, 50 μl) and extracted using Strata × solid phase extraction cartridges (30 mg/ml; Phenomenex, Torrance, CA, USA). Standards and controls were made by dissolving known concentrations of acetaminophen into donor plasma. Acetaminophen and internal standard were separated using a Phenomenex Gemini C18 column (150 × 2 mm, 5 μ). Mobile phase was delivered at a flow rate of 200 μl/min and composed of A (H_2_0 with 0.05% trifluoroacetic acid) and B (acetonitrile:methanol; 50:50). The column was equilibrated with 90%A:10%B and held for 3 min. The mobile phase was increased to 70% B from 3 to 7.1 min, held for 2 min, and then returned to baseline conditions over 30 s and column re-equilibrated. Total run time was 12 min. Acetaminophen (retention time of 6.8 min) and phenacetin (retention time of 8 min) were ionized using an atmospheric pressure chemical ionization source, and parent ions quantitated (152.05 for acetaminophen and 180.10 for phenacetin). The assay was linear from 200 ng/mL to 10 μg/ml, and inter- and intra-assay coefficients of variation (CVs) for control samples at 350, 700, and 7000 ng/ml were <10%. For concentrations below the lowest standard (<3% of samples), samples were re-extracted and reconstituted in a smaller volume and injected onto the LCMS system.

### Gut peptide assessment

Plasma samples for PYY were collected, stored, and assayed following instructions for a commercially available radioimmunoassay (RIA) kit (Millipore, St. Charles, MO, USA; PYYT-66HK), with a sensitivity of 10 pg/ml, and intra-assay and interassay CVs of 5.3% and 7.0%.

### Data analyses

Multi-level models captured within-subject levels and changes in acetaminophen concentrations, gastrointestinal distress, and postprandial PYY (Level 1) and whether group (Level 2) differed in these levels or changes, with random effects for intercept and slope (time) (Keel et al., 2007; Singer & Willett, 2003), using an unstructured covariance matrix. To test whether delayed gastric emptying was associated with binge eating, purging, or both, models were run with the Level 2 effects dummy coded as Binge-Eating Group (0 = Controls and PD *v.* 1 = BNnp and BNp), Purging Group (0 = Controls and BNnp *v.* 1 = PD and BNp), and their interaction – Binge-Eating × Purging Group (0,0 = Controls, 0,1 = PD, 1,0 = BNnp, 1,1 = BNp) under the placebo condition. The same model was run under the medication condition to examine whether increasing gastric motility impacted group differences in gastric emptying and postprandial gastrointestinal distress. Analyses were conducted in IBM SPSS Version 26 with *α* = 0.05.

## Results

### Clinical characteristics

Participants have been described previously (Keel et al., 2018b); however, prior analyses have not evaluated whether clinical features were uniquely associated with binge-eating, purging, or both. Table 1 presents descriptive statistics for each group, and Table 2 presents results from regression analyses in which the main effects of binge-eating group, purging group, and their interaction were tested. Across all variables, there was a main effect of binge-eating group in which those who binged endorsed greater eating pathology, related psychopathology, and impairment compared to those who did not, while controlling for purging group and the interaction of both features. There was also a main effect for purging group in which purging was related to greater eating pathology, related psychopathology, and impairment, while controlling for binge-eating status and the interaction of binge-eating with purging. Finally, there was a significant interaction effect for binge-eating × purging status for all but two clinical features. For eating concerns and depression, there was no additional variance explained by the interaction of binge eating and purging after accounting for their additive effects. For eating concerns, post-hoc comparisons supported significantly higher eating concerns in those with BNp compared to those with BNnp [*t*(42) = 2.10, *p* = 0.04] and PD [*t*(49) = 4.05, *p* < 0.001]; this reflected the presence of both features additively contributing to greater eating concerns in BNp rather than a synergistic effect. The same was true for BDI scores. For the remaining clinical features, the combination of binge-eating and purging explained variance that was not accounted for by the additive presence of both features. However, this interaction indicated *lower* mean scores in those with both binge-eating and purging behaviors than expected by the presence of both features. As an illustrative example, for total EDE scores, the mean (s.e.) difference between those with BNnp compared to controls was 3.34 (0.23) (95% CI 2.71–3.97), and the mean (s.e.) difference between those with PD and controls was 3.20 (0.21) (95% CI 2.62–3.77). However, the mean (s.e.) difference between those with BNp and controls was only 3.99 (0.21) (95% CI 3.41–4.55).

**Table 2.** Clinical features associated with binge eating, purging, and their combination

	Binge eating		Purging		Binge × purge	
Measures	*B* (s.e.)	*t* (91)	*B* (s.e.)	*t* (91)	*B* (s.e.)	*t* (91)
EDE total	5.90 (0.51)	11.49***	5.76 (0.49)	11.84***	−2.56 (0.32)	−8.09***
Restraint	6.44 (0.77)	8.36***	6.79 (0.73)	9.32***	−3.05 (0.47)	−6.43***
Eating concern	2.53 (0.68)	3.71***	1.95 (0.65)	3.02**	−0.58 (0.42)	−1.39
Weight concern	6.65 (0.61)	10.97***	6.34 (0.57)	11.05***	−2.83 (0.37)	−7.58***
Shape concern	6.58 (0.57)	11.65***	6.44 (0.54)	12.04***	−2.92 (0.35)	−8.40***
TFEQ						
Restraint	23.50 (2.79)	8.44***	24.64 (2.64)	9.34***	−11.65 (1.72)	−6.79***
Disinhibition	14.32 (1.80)	7.96***	10.30 (1.71)	6.04***	−4.82 (1.11)	−4.35***
Hunger	9.90 (2.26)	4.38***	6.36 (2.14)	2.97**	−3.25 (1.39)	−2.33*
BSQ	155.50 (15.97)	9.74***	159.33 (15.13)	10.53***	−66.29 (9.84)	−6.74***
BDI	14.06 (4.47)	3.15**	12.86 (4.23)	3.04**	−3.67 (2.75)	−1.33
STAI						
State	26.80 (7.77)	3.45**	26.61 (7.36)	3.62***	−10.90 (4.79)	−2.28*
Trait	29.45 (6.72)	4.38***	26.15 (6.30)	4.15***	−10.50 (4.12)	−2.55*
Impairment						
CIA	31.47 (5.20)	6.05***	25.46 (4.93)	5.17***	−9.68 (3.20)	−3.02**
GAF	−54.34 (3.84)	−14.16***	−56.00 (3.64)	−15.40***	27.07 (2.37)	11.42***

BDI, Beck Depression Inventory (Beck et al., 1961); BSQ, Body Shape Questionnaire (Cooper et al., 1987), CIA, Clinical Impairment Inventory (Bohn et al., 2008); EDE, Eating Disorder Examination (Fairburn & Cooper, 1993); GAF, Global Assessment of Function (First *et al*. 1995); STAI, State-Trait Inventory (Spielberger *et al*. 1983); TFEQ, Three Factor Eating Questionnaire (Stunkard & Messick, 1985).

*p < 0.05, **p < 0.01, ***p < 0.001.

### Gastric emptying

Analyses of gastric emptying under the placebo condition appear in Table 3. The final model supported a significant effect of time [*F*_(1, 94.62)_ = 364.48, *p* < 0.001] and purging [*F*_(1, 95.12)_ = 5.49, *p* = 0.02] with no additional contributions of binge eating [*F*_(1, 95.12)_ = 2.30, *p* = 0.13] or the binge eating × purging interaction [*F*_(1, 95.13)_ = 0.23, *p* = 0.63]. Postprandial acetaminophen concentrations increased significantly over time and were significantly lower in those who purged compared to those who did not, regardless of binge-eating status. The group-level difference indicates that a larger concentration of the test meal had passed from the stomachs to the intestines of those without purging compared to those with purging during the 50 min following consumption. Models that included group × time interaction effects produced worse fit indices, suggesting no differences in the rate of absorption from the intestinal tract to the blood stream once nutrients entered the intestinal tract.

**Table 3.** Associations between gastric emptying, binge eating, purging, and their combination

Parameter	Unconditional growth model		Full model		Best-fitting model	
Conditional likelihood	*b* (s.e.)	95% CI, *p*	*b* (s.e.)	95% CI, *p*	*b* (s.e.)	95% CI, *p*
Intercept	712.41 (7.87)	696.78–728.04, *p* < 0.001	695.24 (11.82)	671.88–718.60, *p* < 0.001	697.34 (10.97)	675.65–719.02, *p* < 0.001
Time (min)	3.25 (0.14)	2.97–3.54, *p* < 0.001	3.22 (0.17)	2.88–3.55, *p* < 0.001	3.22 (0.17)	2.88–3.55, *p* < 0.001
Binge eating			19.61 (13.22)	−6.64 to 45.86, *p* = 0.14	15.36 (9.80)	−4.09 to 34.81, *p* = 0.12
Purging			27.72 (14.56)	−1.18 to 56.62, *p* = 0.06	22.60 (9.80)	3.15–42.05, *p* = 0.02
Binge × purging			−9.39 (19.67)	−48.42 to 29.65, *p* = 0.63		
*Random effects*	*b* (s.e.)		*b* (s.e.)		*b* (s.e.)	
Residual	2257.94 (135.37)	2007.62–2539.48, *p* < 0.001	1440.00 (150.85)	1172.70–1768.20, *p* < 0.001	1440.26 (150.88)	1172.92–1768.54, *p* < 0.001
Intercept	4603.73 (869.72)	3179.10–6666.77, *p* < 0.001	5646.41 (1086.39)	3872.56–8232.78, *p* < 0.001	5633.97 (1083.45)	3864.76–8213.09, *p* < 0.001
Time	0.68 (0.31)	0.28–1.65, *p* = 0.03	1.00 (0.43)	0.43–2.31, *p* < 0.001	1.00 (0.43)	0.44–2.31, *p* < 0.02
BIC	8196.39		4029.61		4023.92	
Deviance (parameters)	8156.66 (6)		3976.34 (9)		3976.57 (1)	
Change in model fit^a^	χ^2^(3) = 490.31, *p* < 0.001		χ^2^(3) = 4180.32, *p* < 0.001		χ^2^(1) = 0.23, *p* = 0.63	

a For the Unconditional Growth Model, change in fit is compared to the Unconditional Means Model (results not included in table) for which BIC = 8686.70, and Deviance (parameters) = 8666.84 (3).

When gastric emptying was measured following administration of a single 10 mg dose of metoclopramide, the only significant predictor of acetaminophen concentrations across models was time (*p* < 0.001 across all models). The presence of purging was no longer associated with gastric emptying in either a model with only main effects [*F*_(1, 95.37)_ = 0.45, *p* = 0.50] or a model that included both main effects and the bingeing × purging interaction [*F*_(1, 95.26)_ = 0.70, *p* = 0.41].

### Subjective responses to the meal

Models predicting gastrointestinal distress supported a main effect for purging [*F*_(1, 83,43)_ = 30.53, *p* < 0.001] and an interaction for bingeing × purging [*F*_(1, 83.35)_ = 6.38, *p* = 0.01] but no significant main effect of bingeing [*F*_(1, 83.40)_ = 1.52, *p* = 0.22]. Those who purged endorsed significantly greater gastrointestinal distress compared to those who did not [*b* (s.e.) = 178.68 (31.39), 95% CI 116.30–241.07, *p* < 0.001], and the presence of binge eating moderated this effect so that gastrointestinal distress was lower in those who binged and purged (BNp) than would be expected based on the presence of purging in this group [*b* (s.e.) = −112.08 (44.36), 95% CI −200.31 to −23.84, *p* = 0.01]. These findings replicate and extend prior findings (Keel et al., 2007, 2018a) by showing a unique elevation of gastrointestinal distress associated with purging in the absence of binge eating in analyses including PD, BNp, BNnp, and controls.

Gastric emptying was a significant predictor of gastrointestinal distress in the placebo condition [*F*_(1, 209.52)_ = 8.60, *p* = 0.004], with lower gastric emptying predicting greater feelings of nausea and stomach ache [*b* (s.e.) = −0.17 (0.6), 95% CI −0.29 to −0.06, *p* = 0.004]. Given that purging was associated with both lower gastric emptying and greater gastrointestinal distress and lower gastric emptying predicted greater gastrointestinal distress in the placebo condition, we tested whether metoclopramide would reduce gastrointestinal distress and eliminate group differences in subjective experiences of nausea and stomach ache.

Participants endorsed *greater* postprandial gastrointestinal distress with medication than without [*F*_(1, 840.14)_ = 7.01, *p* = 0.008; *b* (s.e.) = 22.42 (8.47), 95% CI 5.80–39.03]. Both purging [*F*_(1, 86.43)_ = 28.41, *p* < 0.001] and the interaction of binge × purge status [*F*_(1, 86.34)_ = 11.43, *p* = 0.001] remained significant predictors of gastrointestinal distress following the test meal, with greater distress in those who purged [*b* (s.e.) = 184.69 (30.72), 95% CI 123.83–245.56, *p* < 0.001], that was diminished when combined with binge eating [*b* (s.e.) = −124.22 (43.55), 95% CI −210.59 to −37.86, *p* = 0.005]. There was an interaction between medication and purge group [*F*_(1, 849.07)_ = 3.89, *p* < 0.049; *b* (s.e.) = −6.64 (0.17.28), 95% CI −40.56 to 27.27] that suggested the possibility that the effects of medication on gastrointestinal distress might be lower in those who purged compared to those who did not.

### Exploratory analyses of gut peptide responses

In prior analysis in this sample, we demonstrated significantly greater postprandial PYY release in PD compared to both control and BNp participants under the placebo condition (Keel et al., 2018a). PYY is released from the lower tract of the intestines in response to food ingestion, and its release should be impacted by a medication that increases gastric motility. Consistent with this, higher postprandial acetaminophen concentrations were significantly associated with greater PYY release [*F*_(1, 289.90)_ = 22.93, *p* < 0.001; *b* (s.e.) = 0.16 (0.03), 95% CI 0.09–0.23, *p* < 0.001] and fully eliminated the effect of time in predicting rising postprandial PYY levels [*F*_(1, 198.50)_ ≤ 0.01, *b* (s.e.) ≤ −0.01, *p* > 0.99]. Further, metoclopramide caused significant increases in circulating PYY levels [*F*_(1, 1118.19)_ = 36.84, *p* < 0.001; *b* (s.e.) = 22.81 (3.76), 95% CI 15.43–30.18] and the rate at which PYY increased [*F*_(1, 1123.71)_ = 3.89, *p* < 0.05; *b* (s.e.) = 0.12 (0.06), 95% CI 0.01–0.24].

To understand whether PYY mediated the effects of medication and eating disorder features on gastrointestinal distress, we added PYY to the model along with medication, purging, binge eating, and their interaction. PYY was a significant predictor of greater gastrointestinal distress [*F*_(1, 971.72)_ = 38.33, *p* < 0.001; *b* (s.e.) = 0.37 (0.06), 95% CI 0.25–0.48, *p* < 0.001] and mediated the effect of medication on gastrointestinal distress because the 95% CI of medication included 0 [*b* (s.e.) = 11.17 (15.28), 95% CI −18.82 to 41.16] after including PYY in the model. Even accounting for the effects of PYY on gastrointestinal distress, significant effects of purging [*F*_(1, 86.04)_ = 28.44, *p* < 0.001; *b* (s.e.) = 176.66, 95% CI 116.58–236.75, *p* < 0.001] and the interaction of bingeing × purging remained [*F*_(1, 86.22)_ = 9.39, *p* = 0.003; *b* (s.e.) = −112.86 (42.86), 95% CI −197.87 to −27.85, *p* = 0.01].

## Discussion

We sought to determine whether delayed gastric emptying was associated with purging, binge eating, or both, and results supported a specific association with purging. In addition, delayed gastric emptying was associated with greater postprandial gastrointestinal distress, suggesting that a mechanical disruption in the digestive process may contribute to urges to self-induce vomiting. Combined with prior findings of significantly greater postprandial release of PYY and insulin (Keel et al., 2018a; Maske et al., 2020), findings shed further light on factors that may contribute to the development or maintenance of the core feature of PD. Such information is critical to identifying novel treatment targets. Findings further demonstrated that a single dose of metoclopramide eliminated group differences in gastric emptying. However, it failed to eliminate group differences in gastrointestinal distress. Instead, medication increased gastrointestinal distress in all participants, and exploratory analyses indicated this effect was mediated by the impact of medication on PYY response. Results highlight challenges in isolating a desired outcome from interventions on biological processes with multiple, interconnected consequences.

Very limited research has been dedicated to understanding self-induced vomiting in eating disorders despite the presence of this feature in PD, AN, and BN. Indeed, the DSM-5 eliminated the DSM-IV's distinction between purging and nonpurging BN subtypes due to a lack of empirical support (van Hoeken, Veling, Sinke, Mitchell, & Hoek, 2009). The current study supports biological differences may distinguish those who vomit and extends understanding of this feature beyond traditional explanations of self-induced vomiting as an effort to control weight in a thin-obsessed society.

In addition to providing novel insight into factors that may contribute to purging, current results may explain why efforts to alter gastric emptying proved ineffective in alleviating symptoms in BN. Devlin et al. (2012) conducted a randomized controlled trial to determine the efficacy of erythromycin as a treatment for BN. Prior work had suggested that delayed gastric emptying in BN could account for satiation deficits by contributing to blunted cholecystokinin (CCK) release (Geliebter et al., 1992). Based on this model, a medication that increased gastric emptying should increase CCK response and decrease the risk for large-out-of-control binge episodes. Although patients treated with 500 mg of erythromycin three times daily for 6 weeks demonstrated a significantly faster gastric emptying rate compared to patients in the placebo condition, treatment was not associated with improved clinical outcomes. Current results suggest that gastric emptying may show no unique association to binge eating and that altering gastric emptying rate may be irrelevant to a core symptom of BN – binge eating.

That said, specifying a link between self-induced vomiting and gastric emptying and using a pharmacological manipulation to eliminate group differences in gastric emptying did not open a promising avenue for developing an intervention for PD. Instead, findings provide a cautionary tale regarding the challenges in identifying and isolating a dysfunction within complex systems. Physiologically, metoclopramide increases gastric motility but it also enhances the release of gut peptides that impact feelings of satiation, satiety, and, at high levels, malaise, by increasing how quickly food enters the intestinal tract. In the case of PD, a medication that increases gastric motility may alleviate one cause of gastrointestinal distress while exacerbating another source – exaggerated PYY response. Exploratory analyses identified an association between gastric emptying and PYY release and demonstrated that medication robustly increased PYY response across participants. After the study was funded, the FDA released a black box warning regarding possible serious side effects associated with metoclopramide, which is a dopamine receptor antagonist that crosses the blood-brain barrier and has been linked to tardive dyskinesia. As such, the possible but unlikely anti-emetic benefits appear outweighed by the risks associated with extended treatment. These conclusions align with those regarding the management and treatment of gastroparesis in which delayed gastric emptying causes bloating, excessive fullness, nausea, and vomiting (Camilleri, Parkman, Shafi, Abell, & Gerson, 2013), similar to symptoms observed in PD.

Our novel use of a 2 × 2 factorial design dissociated effects linked to bingeing *v.* purging *v.* their combination. Although prior studies have identified greater pathology in the purging subtype of BN compared to PD or no differences (Smith, Crowther, & Lavender, 2017), this is the first study to demonstrate that clinical differences between purging BN and PD are often smaller than predicted by a simple additive model. This finding introduces some challenges for dimensional models of eating disorders because it suggests that the strength of associations with clinical symptoms changes when symptoms are combined.

Our study enjoyed several strengths. Our rigorous inclusion and exclusion criteria eliminated several common confounds. Our use of a double-blind, placebo-controlled cross-over design permitted stronger inferences regarding group differences in gastric emptying and their downstream effects on subjective and gut peptide responses to food intake. Our measures included structured clinical interview assessments and self-report questionnaires that were selected based on their strong psychometric properties, which were further supported in our current study. Finally, our sample size is the largest in the published literature on gastric emptying in BN, including nearly twice the number of participants included in prior studies, is the first study of gastric emptying in PD, and used a rigorous analytic approach. Moreover, part of this study reflects an independent and successful replication of delayed gastric emptying in purging BN compared to healthy controls.

Despite these strengths, limitations warrant consideration. First, despite our best efforts and a one-year no cost extension, we were unable to secure our target sample of 25 participants with BN who relied solely on nonpurging compensatory behaviors. This produced an unbalanced design in which a majority of those with binge eating were also purging and a majority of those who were not purging were healthy controls. To avoid biased estimates of the main effects of binge eating and purging, we reported results from models with all main and interaction effects and utilized an analytic approach that is well-suited to unbalanced designs. Second, our study utilized a combination of a cross-sectional and experimental design but does not permit causal inferences regarding associations with symptoms. All eating disorder participants were studied during the state of illness and compared to controls who never experienced eating pathology. Thus, we cannot determine whether delayed gastric emptying contributes to self-induced vomiting, is a consequence of self-induced vomiting, or is related to self-induced vomiting through an underlying third variable. Indeed, although our design shares some features with a clinical trial, it reflects a case-control observational design. Prior to comparing groups in the placebo condition, we did not know whether delayed gastric emptying would be associated with bingeing, purging, or their combination. In addition, our use of a single 10 mg dose of metoclopramide was not intended to produce meaningful changes in symptom levels. Instead, it was used to produce its FDA-approved effect on gastric motility to probe downstream consequences on differences in gastric emptying and gastrointestinal distress. We did not employ the current standard, a 4 h scintigraphy of a solid-phase meal, to measure delayed gastric emptying, or standard measures of upper gastrointestinal symptom severity, such as the Gastroparesis Cardinal Symptom Index (Camilleri et al., 2013). Thus, our findings cannot be interpreted as demonstrating gastroparesis in patients who self-induce vomiting to control weight and shape. Finally, our paper focuses on peripheral factors rather than neural systems in the regulation of food intake. Although gastric motility directly influences neural activity via vagal stimulation of the hindbrain, and PYY crosses the blood-brain barrier to bind to receptors in the central feeding circuits, our study did not capture these events or how differences in central responses to peripheral signals might further elucidate clinical features. Likely candidates for observed effects include the area postrema and nucleus of the solitary tract. Understanding the central effects of observed peripheral signals represents an important future direction because activity in these areas feed forward to limbic structures implicated in affective dysregulation which is directly implicated in purging behavior in the absence of binge eating (Haedt-Matt & Keel, 2015).

In conclusion, delayed gastric emptying is linked to purging and may further contribute to gastrointestinal distress described by individuals with PD. Future work may benefit from the use of wireless motility capsule assessment and standard assessments of upper gastrointestinal symptom severity (Camilleri et al., 2013). Such work is needed to determine how best to address this perturbation as efforts to increase gastric motility have demonstrated limited efficacy in those with BN and may do more harm than good in those with PD.
